# Community responses to climate change: Editorial call for submissions to *UCL Open: Environment* Special Series

**DOI:** 10.14324/111.444/ucloe.000028

**Published:** 2021-11-02

**Authors:** Carla-Leanne Washbourne, Sarah Bell, Dan Osborn

**Affiliations:** 1 Department of Science, Technology, Engineering and Public Policy (UCL STEaPP), University College London, London WC1E 6JA, UK; 2 Melbourne Sustainable Society Institute, The University of Melbourne, Victoria 3010, Australia; 3 Chair of Human Ecology, Department of Earth Sciences, University College London, 2 Taviton Street, London WC1H 0BT, UK

## About this series

*UCL Open: Environment* is committed to sharing peer-reviewed knowledge about environmental issues, including climate change. In this special series we are encouraging contributions from authors engaged in producing knowledge about climate change in and with local communities. The published articles and case studies will meet our usual standards of quality and originality, whilst fulfilling the need for wider recognition of transdisciplinary, community-based knowledge.

This special series will include research articles, case studies and commentaries about community-level responses to climate change. We hope that all submissions will be based on work done in collaboration with or led by non-traditional research communities. The special series will provide a platform to record and disseminate the insights from many community-scale and community-led research^1^ [1] projects that are currently poorly visible to researchers and practitioners.

## Climate change and community-scale knowledge

Climate change is arguably the best-known environmental and societal challenge of our time and while calls to respond to the ‘climate emergency’^2^ have been demanded and heeded from international and national decision-makers, many other actions are taking place at smaller spatial scales motivated from and driven by local communities [2,3]. Given its complexity and wide-ranging impacts, responding to the climate emergency requires ‘transdisciplinary’^3^ knowledge, which blurs the boundaries between academic disciplines and recognises and respects different forms of knowledge and expertise [4,5]. Such knowledge is not always easily framed in traditional research papers for publication in discipline-based peer review journals, limiting its sharing between communities, academia, policy and practice.

Local knowledge of climate change impacts, adaptation and mitigation are often overlooked by large research and policy institutions, despite vast potential to contribute to sustainable responses [6]. Where local and indigenous knowledge^4^ [7] are recognised as valuable, they have too often been misappropriated, used without acknowledgement, compensation, permission or benefit to local people and the custodians of cultural knowledge [4,8]. Working with communities opens dialogues and provides different perspectives on how to solve problems (see [9]) that with appropriate recognition and integration can be taken up in either the public or private sphere.

At the same time as awareness of the issues linked to working with communities on environmental topics has grown, scientific knowledge about future climate impacts has also become progressively granular, with more information now available and accessible that is meaningful for local communities [10]. The chair of the UK’s Adaptation Committee recently called on local academics to assist local communities to do their part in mitigating and adapting to climate change (see Event Highlights with respect to the launch of the UK’s Third Climate Change Risk Assessment CCRA3 [11]) setting the scene for further opportunities for knowledge exchange and generation.

## Community responses to climate change

Community-scale and community-based responses to climate change have burgeoned in recent years. In some cases, regions, cities and communities have acted ahead of and in the absence of national and international leadership on key issues [12]. Community energy schemes have demonstrated the democratic and economic potential of the transition to renewable energy [13,14]. Local knowledge is increasingly recognised as important in addressing some issues likely to be exacerbated by climate change, including flood risk management, and local communities have been active in bringing that knowledge in to formal policy and project management processes [15]. Communities that are especially vulnerable to climate impacts, such as those living on the coast, in low-lying areas and in small island states, see a particular urgency to act. They have specific needs for knowledge to support efforts to improve adaptation and increasing examples of local knowledge generation and issue advocacy come from these settings [16,17].

Global governance mechanisms, such as the Paris Agreement [18], provide high-level policy and legal frameworks and allow for discussions amongst major emitters about emission reductions at meetings such as the United Nations Framework Convention on Climate Change Conference of the Parties (UNFCC COP) series, and the G7 and G20 networks. But, even if the world succeeds in limiting global mean temperature increase of 1.5 or 2°C, some climate change impacts are still inevitable and local communities will face difficult choices, including relocation, and other costly decisions. There are questions however about the ability of all our global communities to understand and take action against these impacts at many levels (individual, community, local authority). As the climate justice^5^ movement has pointed out, the people who are most vulnerable to the impacts of climate change are typically those who contribute the least carbon emissions [19,20] making future discussions on accountability, responsibility and capacity for action at different scales necessary and critical.

## Future challenges for community action

The need to respond to the immediate and future impacts of climate change is now widely accepted, and is becoming more urgent. The world is currently about 1°C above the pre-industrial average temperature [21]. This average figure can act to mask extremes of weather and events that are increasingly attributable to climate change such as heatwaves, floods and fires that communities all around the world are already experiencing. Average increases are helpful as a global measure but do not, therefore, reflect what is happening and what will happen at local scales. Some events, such as the recent heatwave that hit communities in the west of the United States are thought by some to be physically ‘impossible’ if it were not for climate change [22,23].

Thanks to a combination of observations and modelling at local scales [e.g., UK Climate Protections (UKCP18)] we know that some communities are facing much higher temperatures than they are accustomed to: the UK is subject to a growing chance of experiencing daily maximum temperatures of over 40°C. [11]. The impacts of climate change will not be evenly distributed across the globe and communities will need to plan and act to be resilient to both short- and long-term impacts and more frequent, unpredictable and perhaps more severe extreme events.

For communities to respond they will probably have to move through several stages of development if they are to help mitigate climate change and also adapt to it. One way that communities may become better able to adapt, and support other communities in the process, is by curating and sharing knowledge and experiences. Little of that has been done so far and doing so might make it easier to:

- overcome barriers to progress that arise from, say, issues linked to laws, governance finance and the psychological frames that people see climate change through and, then, to develop the necessary engineering and behavioural responses, and;
- assist and enable communities to be resilient and thrive, particularly perhaps, those communities facing compound risks (such as those facing flooding from both sea and rivers or extreme heat and drought).

We suspect that some of the most grounded, creative, innovative and transformative work on preparing for and responding to climate change impacts is and will be coming from communities around the world who are themselves foreseeing or currently grappling with the impacts of climate change. Communities of all kinds hold and constantly evolve important knowledge, skills and insights highly relevant to addressing climate change. This special series aims to share knowledge about if and how these are being brought to bear in climate responses in a range of international settings, to understand how communities and researchers might better respond to these coming challenges.

We welcome and look forward to submissions on any aspect of how communities might be addressing climate change as it is a core issue for the world and hope to attract the attention of many actors outside the academic research community as we run up to the COP26 meeting in November 2021.^6^

## Notes

1 Community-scale – for the purposes of this special series we are interested in responses to climate change at or smaller than the scale of local government. We anticipate a focus on communities of place, with an emphasis on civil society and grassroots groups. We welcome other interpretation of ‘community-scale’ and ‘community-led’, within the overall intention of the series to profile work that is under-represented in climate change related academic publishing.

2 Climate Emergency – an increasingly common term used to describe the fact that climate change poses an immediate and substantive threat to human life and the biosphere (e.g., [1]).

3 Transdisciplinarity – an approach to knowledge generation which exhibits: “[a] focus on ‘wicked problems’ that need creative solutions … reliance on stakeholder involvement, and engaged, socially responsible science … simultaneously studying multiple levels of, and angles on, reality” (e.g., Bernstein 2015).

4 Indigenous and local knowledge – ‘knowledge and know-how accumulated across generations, which guide human societies in their innumerable interactions with their surrounding environment’ [7].

5 Climate justice – an emerging term that seeks to include, in the context of climate change: ‘local impacts and experience, inequitable vulnerabilities, the importance of community voice, and demands for community sovereignty and functioning’.

6 https://www.gov.uk/government/news/holy-see-faith-and-science-an-appeal-for-cop26.
